# Feeding-Triggered Seizures in a Newborn with *AP1S1*-Related MEDNIK Syndrome: Expanding the Phenotype of a Hyper-Rare Disease

**DOI:** 10.3390/jcm15010106

**Published:** 2025-12-23

**Authors:** Anna Cavalli, Francesca Peluso, Daniele Frattini, Carlo Alberto Cesaroni, Carolina Bondi, Giovanni Malmusi, Adelaide Peruzzi, Susanna Rizzi, Agnese Pantani, Gabriele Trimarchi, Nives Melli, Antonio Novelli, Roberta Zuntini, Giancarlo Gargano, Livia Garavelli, Carlo Fusco

**Affiliations:** 1 Child Neurology and Psychiatry Unit, Arcispedale Santa Maria Nuova, AUSL-IRCCS di Reggio Emilia, 42123 Reggio Emilia, Italycarloalberto.cesaroni@ausl.re.it (C.A.C.); susanna.rizzi@ausl.re.it (S.R.); agnese.pantani@ausl.re.it (A.P.);; 2 Medical Genetics Unit, Santa Maria Nuova Hospital, AUSL-IRCCS di Reggio Emilia, 42123 Reggio Emilia, Italy; francesca.peluso@ausl.re.it (F.P.); gabriele.trimarchi@ausl.re.it (G.T.); roberta.zuntini@ausl.re.it (R.Z.);; 3 Neonatal Intensive Care Unit, Arcispedale Santa Maria Nuova, AUSL-IRCCS di Reggio Emilia, 42123 Reggio Emilia, Italy; 4 Laboratory of Medical Genetics, Translational Cytogenomics Research Unit, Bambino Gesù Children’s Hospital IRCCS, 00165 Rome, Italy

**Keywords:** neonatal seizures, MEDNIK syndrome, *AP1S1*, IDEDNIK, feeding-triggered seizures, metabolic epilepsy, copper metabolism disorders, Elesclomol

## Abstract

MEDNIK syndrome (Mental Retardation, Enteropathy, Deafness, Neuropathy, Ichthyosis and Keratodermia) is a severe hyper-rare condition resulting from the biallelic variants in the *AP1S1* gene, implicated in intracellular trafficking and copper homeostasis. Only 18 affected individuals (seven *AP1S1* pathogenic variants overall) have been reported to date, with a high early lethality due to life-threatening congenital enteropathy. Seven patients have been empirically treated with zinc. Due to the paucity of literature data, little is known about the clinical course of individuals affected by MEDNIK syndrome, and the possible early association with epilepsy needs to be investigated. We present the first case of Italian origin affected by MEDNIK syndrome carrying a new homozygous *AP1S1* stop variant, presenting with congenital severe enteropathy and feeding-related seizures, thus representing an early, singular manifestation of the disease. We describe her clinical course and the zinc acetate therapeutic experience. We also reviewed the literature focusing on clinical manifestations (especially neurological), brain neuroimaging and the symptom evolution of patients with *AP1S1*-related MEDNIK syndrome and discuss possible future therapeutic attempts.

## 1. Introduction

MEDNIK syndrome (Mental Retardation, Enteropathy, Deafness, Neuropathy, Ichthyosis and Keratodermia) is a hyper-rare autosomal recessive disease with multisystemic involvement, caused by biallelic variants in the *AP1S1* gene [1]. First described in 2005 as a neurocutaneous disease [2,3], it was later listed as a disorder of copper metabolism [4].

Recently, MEDNIK syndrome has been listed as IDEDNIK (Intellectual Disability, Enteropathy Deafness, Peripheral Neuropathy, Ichthyosis and Keratoderma) syndrome, together with KIDAR (Keratitis-Ichthyosis-Deafness Autosomal Recessive) syndrome, also known as MEDNIK-like syndrome, due to *AP1B1* gene mutations [5]. These two hyper-rare conditions are characterized by clinical and pathogenetic similarities. Despite the paucity of cases, for greater phenotypic clarity, in this paper we will review patients with *AP1S1*-related MEDNIK syndrome only.

The *AP1S1* gene encodes the small subunit σ1A of the Adaptor Protein 1 (AP1) complex, a clathrin-coated assembly that plays a crucial role in trafficking between the trans Golgi network, endosomes and the plasma membrane. Adaptinopathies are an emerging group of diseases caused by defects in AP complexes (AP1–5), which share the propensity to affect organs of neuroectodermal origin, with a variable degree of severity [6]. Although the pathogenic mechanisms of MEDNIK syndrome are complex and not yet fully elucidated, it has been shown that defects in *AP1S1* affect the intracellular localization of copper pump *ATP7A* (and probably also *ATP7B*), impact mitochondrial function and indirectly perturbate peroxisomal metabolism [4].

From a biochemical perspective, MEDNIK syndrome shares certain signs with the two best-known copper metabolism disorders, Menkes and Wilson’s diseases. In Menkes disease, defects in the *ATP7A* pump result in impaired copper absorption in the gut and decreased copper passage through the blood–brain barrier; both plasma levels and urinary excretion of copper are low, as is plasma ceruloplasmin. In Wilson’s disease, defects in the *ATP7B* pump result in impaired excretion of copper by the liver into bile, leading to tissue copper overload, mainly in the liver and brain; plasma ceruloplasmin levels are usually low, while urinary copper excretion increases and the copper values, typically increased in liver tissue, may be normal or low in plasma due to reduced ceruloplasmin [7,8]. Patients with MEDNIK syndrome exhibit reduced concentration of both plasma copper and ceruloplasmin, while their urinary copper excretion is increased, with hepatic copper accumulation documented through liver biopsy [4,9,10,11,12]

To date, only 18 patients with *AP1S1*-related MEDNIK syndrome have been reported overall, with a high early lethality. Clinical manifestations include severe congenital enteropathy, deafness, cholestatic hepatopathy, peripheric neuropathy, hypotonia, neurodevelopmental delay and skin involvement [2,3,4,9,10,11,12,13].

Seizures were briefly mentioned in three case reports [10,12,13]; however, the occurrence of feeding-triggered seizures have not been reported. Few patients have been empirically treated with zinc, with little or no information on clinical course [4,6,9,10,11].

We report a neonate of Italian origin with MEDNIK syndrome carrying a new homozygous *AP1S1* premature stop codon variant, presenting with congenital severe enteropathy and feeding-related non-motor seizures as the early manifestations of the disease. We also describe her clinical course and the zinc acetate therapeutic experience and review the literature focusing on neurological aspects and symptom evolution.

## 2. Detailed Case Description

The female patient is the third child born to healthy parents of Italian origin, unknowingly consanguineous, who come from neighboring villages in Campania, just 15 km apart.

Family history is unremarkable except for early dilated cardiomyopathy on the paternal line and psoriasis on maternal line. Pregnancy proceeded with gestational diabetes mellitus and polyhydramnios. The patient was born at 37 weeks of gestation, from vaginal delivery. The Apgar Index was 9/10 at the 1st min and 10/10 at the 5th min, weight at birth was 2.955 kg (50th centile, −0.02 SDS), length was 50 cm (80th centile, 0.89 SDS) and occipital frontal circumference (OFC) was 33 cm (35th centile, −0.34 SDS).

Since birth, the patient presented with severe, persistent, noninfectious diarrhea, while her clinical examination, including tone, alertness and sucking reflex was normal. Initially, the baby was fed breastmilk. Due to progressive dehydration and weight loss, at 5 days of life she was admitted to Neonatal Intensive Care Unit (NICU) and she was fed aminoacidic formula; however, there was no improvement, since the diarrhea persisted. At 15 days of life, enteral feeding was terminated, and total parenteral nutrition (TPN) was established, with consequent clinical rapid improvement and weight gain.

At 22 days of age, a first enteral refeeding attempt failed due to the recrudescence of the enteropathy and the simultaneous onset of focal non-motor seizures, with prominent autonomic manifestations characterized by skin pallor, behavior arrest and hypoventilation, which resolved upon reinstating TPN.

**Figure 1:**
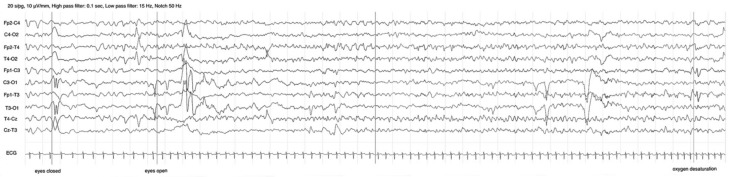
Feeding-triggered seizure EEG recording (the patient was 35 days old): focal epileptic discharge arising with left temporal onset and rapid bilateral spreading. Clinically, the child suddenly opens her eyes and exhibits a brief, isolated, widespread myoclonus; then, she presents behavioral arrest and autonomic manifestations (pale skin and hypoventilation with rapid drop in plasma oxygen saturation). The seizure lasts two minutes, then resolves, and the child returns to normal responsiveness. See also the video-EEG seizure recording in Supplementary Materials.

Over the next two months, a further four refeeding attempts were carried out, but they all failed within 24 h due to the onset of focal seizures lasting 1–2 min, as documented by video-EEG recording (Figure 1, Supplementary Materials Video S1).

Seizures presented in clusters (from three to nine seizures in 24–48 h, without configuring an epileptic status). They were fairly similar in terms of semeiology and duration, characterized by a predominantly non-motor phase with arrest and hypoventilation, although brief diffuse myoclonic jerks were occasionally observed. Seizures were time-related to enteral feeding, and they resolved upon reinstating TPN. There was no concurrent electrolyte imbalance nor significant acid–base disfunctions, except a mild hyperlacticaemia (max value 42 mg/dL with normal range 4.5–19.8) during the second seizure’s cluster.

During the first seizure cluster, she was treated with phenobarbital (PB) i.v. 20 mg/kg once, followed by PB 5 mg/kg/day for 5 days only. Then, starting from the second seizure cluster, oral carbamazepine (CBZ) was introduced, up to 15 mg/kg/day. Antiseizure medication (ASM) did not modify the occurrence of seizures, which instead resolved by interrupting enteral nutrition.

We also observed a questionable mild worsening of background EEG activity while attempting enteral refeeding, consisting of bilateral slow rhythm surplus, during booth sleep and awakening EEG recordings, not attributable to post-critical activity.

Biochemical metabolic investigations were unremarkable: we documented a transient mild metabolic compensated acidosis (−10 base excess at arterial blood gas analysis) and hyponatremia (lowest value 131 mmol/L). Plasma amino acids, acylcarnitine, vitamin B12, homocysteine, lacticaemia and ammonium were in the normal range. We performed a lumbar puncture, revealing normal Cerebrospinal Fluid (CSF) values, including pressure, appearance, total protein, glucose, gamma-globulin and lactate. Inflammation markers and infectious tests on plasma, urine and stool were all negative.

A mild congenital bilateral hearing loss was documented, while eye investigation and early abdomen ultrasound were unremarkable. Cardiac ultrasound revealed *patent foramen ovale* (PFO) without hemodynamic changes.

**Figure 2:**
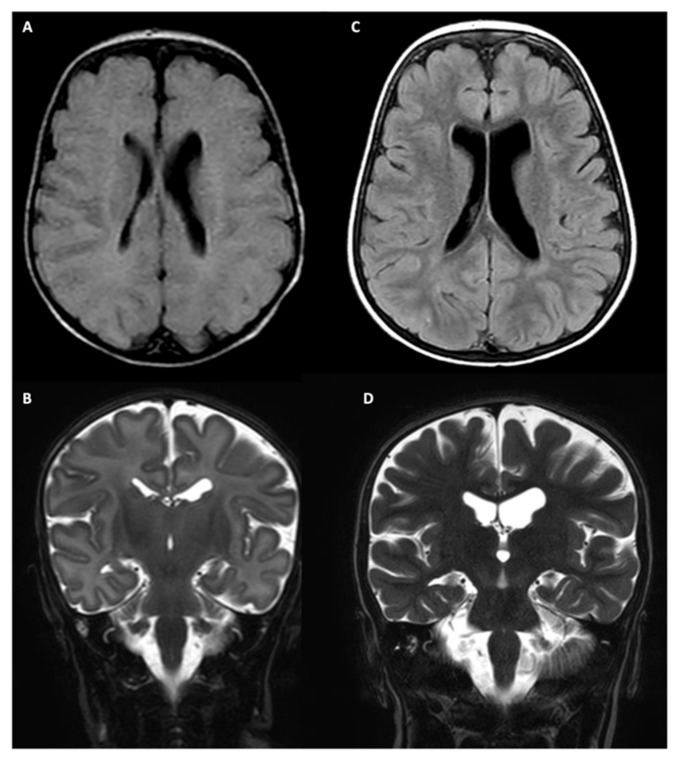
Brain MRI: (**A**,**C**) axial FLAIR; (**B**,**D**) coronal T2 sequencies. The patient’s brain MRI, performed at the age of 1 month, showing mild asymmetry of lateral ventricles; brain MRI of the same girl repeated at the age of 33 months, showing atrophic progression mainly affecting the white matter and the hippocampi.

A brain MRI, first performed at the age of 1 month, revealed no abnormalities, except for a mild asymmetry of the lateral ventricles (Figure 2A,B). At the age of 33 months, MRI showed bilateral volume loss and incomplete inversion of the hippocampi, as well as bilateral hippocampal hyperintensity on T2-weighted and FLAIR sequences. The white matter was diffusely thinned, while ventricular and peri-encephalic spaces were widened (Figure 2C,D).

Cholestatic hepatopathy emerged at the age of two months: biliar acids 10.7 mmol/L (n.v. 20–60), Alanine transaminase (ALT) 45 U/L (n.v. < 40), Aspartate transaminase (AST) 50 U/L (n.v. < 49), Gamma-glutamyl transferase 78 U/L (n.v. < 38).

The case was considered worthy of genetic investigations. CGH-SNP array analysis revealed multiple regions of homozygosity (2%). A subsequent clinical exome analysis identified the homozygous c.256C>T p.(Arg86Ter) variant (rs1475768696) in the *AP1S1* gene (NM_001283.5), resulting in a premature stop codon in exon 3 (out of 5). This variant is predicted to cause loss of normal protein function through either truncation or nonsense-mediated mRNA decay and is not described in the literature, but it is present in gnomAD v.4.1.0 (accession 9 September 2025) with an allelic frequency of 0.000001271. The parents were both heterozygous for the variant. According to the American College of Medical Genetics and Genomics/Association for Molecular Pathology (ACMG/AMP) recommendations [14], the variant was classified as likely pathogenic [PVS1, PM2]. We confirmed the diagnosis of MEDNIK syndrome.

As an incidental finding, the missense variant c.11708G>A p.Arg3903Gln (rs148399313) in the *RYR1* (NM_000540.3) gene inherited by the mother emerged. This variant is present in gnomAD v.4.1.9 (accession 9 September 2025) with an allelic frequency of 0.000009293. This variant has been reported in several individuals and families affected with malignant hyperthermia susceptibility [15,16,17,18] and was classified as likely pathogenic on 6 April 2023 by the ClinGen Malignant Hyperthermia Susceptibility Variant Curation Expert Panel (ClinVar ID 133017) [19]. To our knowledge, functional studies have not been reported for this variant, but computational prediction tools and conservation analyses suggest that this variant may impact the protein RYR1. According to ACMG/AMP recommendations [14], the variant was classified as likely pathogenic [PS4, PM2, PP3, PM5].

Based on the few published studies, zinc acetate supplementation was introduced at the age of 2 months. The dosage was increased up to 3 mg/kg/day [4,9].

Although seizures were clearly triggered by enteral refeeding, at the age of three months, weaning from TPN became a clinical priority. Thus, it was decided to tolerate the few seizure clusters in order to reintroduce enteral nutrition. The patient was gradually weaned off TPN, and from 4 months onwards, she was only enterally fed, carrying a permanent central venous catheter (CVC) in case of emergency. Seizures persisted until the age of 6 months, tending to present in clusters (1–2 clusters/month), clinically characterized by early, predominantly non-motor features (behavior arrest, pale skin and hypoventilation), followed by a brief tonic phase with cyanosis.

ASM with CBZ was continued, and plasmatic CBZ dosages were in the therapeutic range. At 6 months of age, seizures ended spontaneously, while EEG documented poor background activity maturation. From 6 months onwards, she remained seizure-free while taking ASM.

At 10 months of age (after 8 months of zinc therapy), the patient manifested severe hyporegenerative anemia, requiring blood transfusions and neutropenia: neutrophiles 140/microl (n.v. 1600–7500). Zinc acetate was discontinued due to its possible causative role in anemia genesis [20,21,22,23]. Moreover, out of caution, CBZ was also switched to levetiracetam (LEV), and the patient stayed seizure-free. Hematological problems fully resolved, but in agreement with the child’s parents, we decided not to reintroduce zinc for at this time.

In Table 1 we report the copper metabolism and hepatic function biochemical values before, during and after the zinc treatment. A liver biopsy was not performed, as it was deemed inappropriate given that the diagnosis was certain and the procedure would not offer any therapeutic benefit despite being invasive.

As expected, plasmatic copper and ceruloplasmin, already low before starting zinc, were suppressed after 8 months of therapy and returned to baseline (low) values after zinc discontinuation.

**Figure 3:**
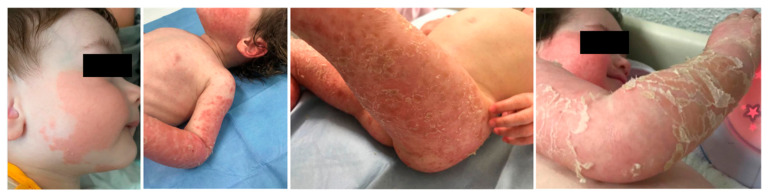
Skin involvement: relapsing-remittent erythematous-desquamative plaques, with skin folds worsening.

At 7 months of age, dermatological involvement became evident, characterized by relapsing-remittent erythematous-desquamative plaques, with skin folds worsening, compatible with the underlying diagnosis (Figure 3).

At the age of 18 months, electroneurography (ENG) was performed, showing peripheral nerve conduction within the normal range. ENG was performed two more times over time, at ages two and three years and seven months respectively, revealing no abnormalities.

Her development indicated a moderate psychomotor delay, mainly affecting motor function, while cognitive and relational competences were just mildly impaired. Sitting position and babbling were both acquired at the age of 18 months. At last examination, the patient was 3 years and 7 months of age; she displayed severe axial hypotonia and ligament hyperlaxity, she still did not walk, neither language was acquired, but she manifested relational interest and vocalized with communicative intent. No behavioral concerns were reported by the parents. Her auxologic parameters fit the normal range, but they were quite below average values (weight at 18th centile, −1.2 SD; height at 5th centile, −1.8 DS).

She presented mild chewing and swallowing impairment and thus her diet consisted of soft food divided into small pieces.

Considering the prolonged seizure-free period, LEV was gradually stopped at the age of 3 years.

**Figure 4:**
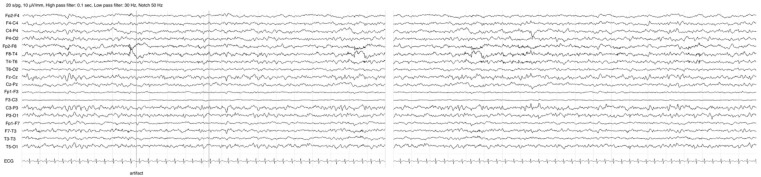
EEG evolution: the patient’s awake background activity at last follow-up at 3 years and 7 months, showing slowed background activity and theta waves in bilateral central regions without epileptic discharges.

The last EEG examinations (3 years and 7 months of age) showed a mildly slowed background activity during the awake state, theta waves in bilateral central region and poor sleep-figure organization, without epileptic discharges (Figure 4).

## 3. Discussion

We describe a new case of MEDNIK syndrome, presenting with congenital enteropathy and clusters of feeding-related seizures that first manifested at the age of 22 days, thus representing an early key feature of the clinical picture. Later, as expected, other syndrome hallmarks, such as cholestatic hepatopathy, erythrokeratodermia, ichthyosis, growth retardation, hypotonia and psychomotor delay, emerged.

To date, only 18 individuals with *AP1S1*-MEDNIK syndrome have been reported [2,3,4,9,10,11,13]; thus, it must be considered a hyper-rare syndrome, according to Smith’s classification [1]. Our case raises to 19 the overall number of patients.

Due to the paucity of cases and the high neonatal lethality, little is currently known about the frequency of symptoms and the natural history of the disease. Furthermore, the early association with epilepsy has not yet been detailed and needs to be investigated.

We reviewed the literature on *AP1S1*-related MEDNIK syndrome, focusing on neurological features, clinical, molecular, biochemical and neuroradiological hallmarks, therapeutic attempts and disease course (Table 2, Supplementary Materials Table S1).

Among the 18 affected individuals reported to date, eight patients (the Canadian cohort) belong to five families, which are likely to share common ancestors [2,3,4]. The remaining subjects are spread globally: two unrelated Turkish cases [9,13], four Romans carrying the same genotype [11,13], one from Mexico [10], two Chinese siblings [12] and one patient with Sephardic Jewish origin [4]. To our knowledge, our case is the first native Italian MEDNIK patient. There is a female gender prevalence (15/19); in one case (patient KEK V04-03 of the Canadian Cohort) the gender is not reported [3].

All 19 patients reported overall (including this case) display of homozygous *AP1S1* variants. No affected individual with *AP1S1* compound heterozygous variants has been reported to date. Due to the founder effect, the 18 previously reported patients, which derive from a limited number of ethnic groups, carry a total of seven pathogenic variants: two missense, two truncating, one splicing and two frameshift. Our patient displayed a new homozygous *AP1S1* variant, never described before, classified as likely pathogenetic and predicted to cause loss of normal protein function through either truncation or nonsense-mediated mRNA decay. This case raises to eight the *AP1S1* pathogenic variants reported worldwide.

The patient and her mother present also, as incidental findings, a likely pathogenic *RYR1* variant, already described in the literature in many individuals affected with malignant hyperthermia susceptibility, a potentially life-threatening, inherited muscle disorder that causes a rapid, uncontrolled metabolic reaction in susceptible individuals exposed to certain anesthesia drugs and triggers. This information allows anesthesiologists to avoid certain anesthesia drugs in the case of surgery.

In all but one *AP1S1*-related MEDNIK reported case, severe congenital enteropathy represented the first symptom, characterized by watery stool, sometimes with a transient blood component, often causing life-threatening dehydration and requiring admission to the NICU.

It is unknown what protective factors, if any, prevented patient #1 in Duan’s report from developing neonatal diarrhea. He appears to be the only patient with *AP1S1*-related MEDNIK syndrome who did not present with this symptom at onset, unlike his affected sister [12].

Based on the literature review, we evaluate an overall mortality rate (MR) for *AP1S1*-related MEDNIK syndrome of 58% (11/19). Nine out of eleven patients died before the age of two years, all of them because of enteropathy: three individuals during their first month of life [2,13], four between one month and one year of age [2,3,11,13] and two patients between their first and second birthday [3]. Only two patients died after the age of two, at 5.5 years, due to aspiration in home care [11], and 27 years of age (original id patient KEKV 02-03), without a reported cause [2]. The MR is higher if considering the Canadian cohort only (MR 75%) and lower in the most recently reported patient group (MR 45%). We speculate that this MR gap could reflect a disparity in the experimental neonatal intensive care practice for Canadian patients, born before 2000 or in the early 2000s, compared to the cutting-age techniques available for the second group of patients, more recently born. Again, it cannot be excluded that the Canadian cohort genetic pedigree determined a more severe phenotype. Finally, the overall mortality rate may be underestimated because of the limited information available about the patients’ clinical course.

Our patient, like the others, presented with congenital enteropathy. Furthermore, she also experienced clusters of feeding-related neonatal seizures, constituting an early, pivotal clinical feature. We observed a clear time correlation between refeeding attempts and the onset of seizures, which occurred a few hours (<24 h) after enteral food intake, even when taking a minimal amount of different milk formulas (hydrolyzed, amino acid-based, etc.).

The advent of seizures in MEDNIK syndrome has been reported in four patients to date, but the specific role of nutrition in triggering seizures, as it happened in our patient, has not been described before. Klee et al. briefly mentioned an episode of myoclonic seizures responsive to phenobarbital during severe sepsis, leading to patient death [13]; Lu at al. report a neonate with seizures treated with phenobarbital in the course of metabolic acidosis, lethargy, electrolyte imbalance and acute kidney failure [10]. In both of these cases, seizures seemed to occur in neonates during severe metabolic acidosis, and they had a monophasic course. In a recent paper by Duan et al. [12], two siblings with seizures are reported: case #1 experienced convulsive seizures at the age of two months and received levetiracetam for the following 4 years, being seizure free; his young affected sister (case #2) was reported to experience a cluster of seven convulsive seizures in a single day, lasting approximately 1 min each, at the age of two months, while diarrhea was occurring, but no information is provided on the type of nutrition being taken at that time. She received levetiracetam for the next 7 months; the parents discontinued this treatment, but at the age of 2.2 years seizures recurred, requiring valproic acid adjunct to levetiracetam to control them.

In our case, the epilepsy phenotype was crucial in suspecting a metabolic disease instead of other sources of genetically based congenital diarrhea or a severe allergy to cow’s milk proteins, thus leading to the prompt molecular diagnosis. Our patient manifested seizure without simultaneous electrolyte alterations, acid–base imbalance, or hyperammonemia. A mild isolated hyperlacticaemia was found in the course of the second cluster of seizures, maybe due to the occurrence of the seizures themselves. CSF values, including CSF lactate, were in the normal range, although we did not perform a lumbar puncture in the context of seizure clusters. Seizure recurrence and the clusters’ frequency seemed to be unaffected by ASM, as they were triggered by enteral refeeding attempts and ended spontaneously at 6 months of age, while EEG documented poor background activity maturation.

The literature lacks evidence on the epilepsy course and epilepsy itself in MEDNIK syndrome. In our case, the occurrence of feeding triggered seizures, unreported before in any disease of copper homeostasis including Menkes disease, seems to resemble the epilepsy phenotype in some mitochondrial conditions.

More than one pathogenetic mechanism of epilepsy in MEDNIK syndrome can be hypothesized. First of all, investigations into MEDNIK syndrome patients’ fibroblasts document a marked reduction of Cytochrome oxidase (COX) II, COX-IV and superoxide dismutase, thus perturbating mitochondrial function [4]. It can therefore be supposed that the intracellular metabolic stress induced by enteric carbohydrate intake could be a seizure trigger in this condition. Secondly, a deficit in cuproenzymes (such as dopamine-beta-hydroxylase) interferes with neurotransmitter biosynthesis. Lastly, perturbations in systemic copper levels and imbalances in intra-extracellular copper concentrations perturbate the synaptic function acting on NMDA, GABAA and AMPA receptors, thus resulting in disturbance of the neuronal excitatory–inhibitory balance [24,25,26]. Similar mechanisms are likely implicated in the epileptogenesis of Menkes syndrome, where drug-resistant epilepsy is a recognized key symptom [27]. Interestingly, seizures can also be triggered by irregular use of copper chelation drugs in patients with Wilson’s disease [21,22,28,29,30,31]. In *AP1S1*-MEDNIK syndrome, the copper homeostasis perturbation is sustained by multiple mechanisms, and its relation to epileptogenesis is yet to be clarified. Although many further studies are needed, the evidence of feeding-triggered seizure could add insight into the pathogenetic mechanism awareness of such a complex metabolic condition.

Starting from the age of 2 months, our patient progressively displayed other hallmarks of the condition, such as cholestatic hepatopathy, erythrokeratodermia, ichthyosis, early growth retardation, hypotonia and psychomotor delay. Taking an overview of the clinical features reported in affected patients to date (Figure 1, Supplementary Materials Table S1), cholestatic hepatopathy, hypotonia and growth retardation seem to represent constant features, while deafness and neuropathy may not be present. Skin involvement is reported in most patients, characterized by erythematous patches evolving into erythro-desquamative lesions and ichthyosis, with a fluctuating trend. Facial dysmorphic signs are described in a few individuals belonging to the Canadian cohort (high forehead, triangular face) and in two Chinese siblings (high forehead, sparse teeth, hair and skin hypopigmentation, nail abnormalities). Although further evidence is needed, the finding of hypopigmentation of hair and skin, as in Menkes disease, requires attention. Contrarily, other dysmorphic traits could be related to a specific familiar lineage rather than the condition itself.

According to Alsaif and Alkuraya [5], the clinical picture of MEDNIK syndrome seems to be quite specific, considering that symptom frequency can be underestimated due to early high lethality. Moreover, the few available cases are published in different scientific journals, each focusing on specific clinical features of the condition, which leads to a deeply multisystemic presentation.

Like all other reported patients whose data are available, our patient presents moderate motor delay associated with severe hypotonia as well as language delay. All *AP1S1*-related MEDNIK patients seem to display a psychomotor delay with some degree of cognitive impairment; autistic features are reported in one case [4], and one child was diagnosed with attention deficit hyperactivity disorder [12]. Furthermore, it is reasonable to suppose that other children with this condition experienced dysphagia, but there is a lack of literature on this topic.

Our patient’s brain MRI at the age of one month revealed no abnormality, while over two years later, we detected a volume loss, mainly involving the hippocampi and white matter and hippocampal structural abnormalities (incomplete inversion and long-TR hyperintensity). Among the previously reported data, brain MRI data are reported in 8/18 patients [2,3,4,9,12]: among them, six patients (75%) displayed brain atrophy and three of them also presented a mild basal ganglia T2-hyperintensity, although information on the patient’s age at the time of the investigation is not available. In the two sibling patients reported by Duan et al. [12], the brain MRI was normal.

The atrophic cortical evolution we detected by performing brain MRI scans over time, never detailed before, confirms the progressive and neurodegenerative course of MEDNIK syndrome, as suspected based on the pathogenetic mechanisms. Our case shows a prevalent bilateral hippocampal involvement, which has not previously been reported, while the basal ganglia are not specifically targeted. Analyzing the literature, we observe that all three patients who displayed basal ganglia abnormalities are still alive or died in young adulthood; thus, we hypothesize that brain MRI could have been performed during late childhood instead of at a neonatal/toddler age, and consequently we speculate that basal ganglia hyperintensity could appear in a second stage of the disease. We plan to repeat the brain MRI in our patient around the age of 6–8 years to evaluate if basal ganglia involvement has developed over time.

The same consideration can be made for peripheral neuropathy, which is mentioned in 7/11 (64%) of previously reported cases whose data are available [2,11,12]. Although we performed electroneurography annually, our patient has shown no signs of neuropathy to date. The peripheral nervous system may not always be involved, or it may be a sign that appears later, as the condition progresses.

In the literature, seven patients were empirically treated with zinc, with no homogeneity in drug formulation and doses: four individuals received zinc acetate, one zinc solphate and two zinc gluconate granules. Inconspicuous improvement is reported in two cases, mainly regarding cholestatic hepatopathy and cognitive function, while dermatological symptoms and enteropathy had no benefit. The remaining had no or unclear benefits [4,6,9,10,11]. As soon as we received the molecular diagnosis, zinc acetate treatment was started. The dosage was increased up to 3 mg/kg/day, which corresponds to usual zinc acetate dosage in pre-symptomatic children with Wilson’s disease. We cannot say with certainty whether our patient actually benefited from the treatment, since she did not experience any clinical worsening nor any clear improvement that was time-related to the zinc consumption; in addition, there is no literature data on the natural history that allows a comparison. In general, as the child grew, we observed an improvement in liver function and nutrition but a stagnation in psychomotor development. We were aware that, theoretically, the zinc treatment could paradoxically exacerbate symptoms related to copper deficiency in patients with MEDNIK syndrome, including possible hematological and neurological consequences [21,24,26]. However, since it was the only therapy available, we decided to start treating the patient and monitor her closely.

We wondered whether it might make sense to empirically combine zinc therapy with low-dose copper supplementation in order to balance the effects of the former. We consulted with experts in metabolic diseases, but the lack of previous therapeutic attempts with copper in combination with zinc in MEDNIK syndrome led us to err on the side of caution.

During zinc treatment, our patient experienced severe hyporegenerative anemia and severe neutropenia. Zinc supplementation is known to cause anemia in Wilson’s disease patients; however, any possible hematological involvement in MEDNIK syndrome that predisposes to this eventuality should be investigated in the future [20,21].

Recently, the mitochondrion-targeting antitumoral Elesclomol has been proposed in copper metabolism disorders [32,33]. It is definitely worth looking at the preliminary results of the experimental use of the drug in two children with Menkes disease, as it seems to be beneficial, especially concerning central nervous system involvement [34]. In this study, the two children, who received a Menkes diagnosis at birth, were given weekly Elesclomol subcutaneous injection adjunct to daily copper histidine treatment. One child started Elesclomol at the age of 20 months when already displaying neurodevelopmental deficits; the second child started at 2 months of age, while his neurodevelopment was in normal range. Standardized assessments documented a significant improvement in the first patient, and the maintenance in the second, of cognitive, motor and communication performance. Menkes disease and MEDNIK syndrome are certainly two different conditions, but they share some similarities in clinical presentation, biochemical parameters and pathogenetic aspects. Therefore, these findings are also interesting for their potential future implications for patients with MEDNIK syndrome.

## 4. Conclusions

We report an Italian native newborn girl with MEDNIK syndrome due to a new *AP1S1* variant, presenting with feeding-triggered seizure clusters, which constituted a pivotal undescribed onset symptom.

MEDNIK syndrome is a complex, multisystemic and progressive condition with a high mortality rate. We would like to emphasize that MEDNIK syndrome should be strongly considered in newborns with intractable, life-threatening diarrhea and feeding-triggered seizures, even in the absence of other key symptoms, which may appear later. This is particularly important when consanguinity is declared or suspected.

As non-motor seizures can be difficult to detect, we recommend carrying out EEG investigations on patients suspected of having this condition.

MEDNIK syndrome has no approved treatment to date. The paucity of reported cases and the lack of studies on the natural history could hinder possible efforts in developing precision therapy in the future. The establishment of an international registry of MEDNIK syndrome would be very beneficial for physicians and researchers.

## Figures and Tables

**Table 1:** Plasmatic values of copper homeostasis and liver function over time: ceruloplasmin, copper, zinc, alanine-aminotransferase (ALT), aspartate-aminotransferase (AST) and gamma-glutamyl-transferase (gamma GT) values of the patient prior to, during and after zinc therapy.

Plasmatic Values	Before Zinc Therapy (Age 2 Months)	Eight Months of Zinc Therapy, Ongoing (Age 10 Months)	After Zinc Withdrawal (Age 12 Months)
Ceruloplasmin (g/L) n.v. 0.22–0.58	0.14	0.02	0.12
Copper (μg/dL) n.v. 50–150	35	3	29
Zinc (μg/dL) n.v. 13–40	175	150	120
AST (U/L) n.v. 2–40	50	51	40
ALT (U/L) n.v. 4–49	90	81	85
Gamma GT (U/L) n.v. 2–38	38	81	57

**Table 2:** Summary of *AP1S1*-related MEDNIK genetic and clinical features: review of genetic and clinical features of all reported *AP1S1*-related MEDNIK cases (including ours), focusing on neurological manifestation of the disease and therapeutic attempts (extensive table included in Supplementary Materials); *n* = number; *n*/tot available data = number of patients presenting this feature/total number of patients for whom data on this feature is available; (*a*) = patients are also described by Montpetit [3] and Martinelli [4]; (*b*) = two patients are also reported by Martinelli [4]; (*c*) = Duan [12] does not report the occurrence of congenital enteropathy in case #1 despite a detailed clinical description.

*Original Paper*	*Saba 2005* [2] *^(a)^*	*Montpetit 2008* [3] *^(b)^*	*Martinelli 2013* [4]	*Incecik 2018* [9]	*Klee 2020* [13]		*Lu 2023* [10]	*Rackova 2024* [11]	*Duan 2025* [12]	*Our case*	Total
*n* Reported patients	5	3	1	1	3		1	2	2	1	19
*Origin*	Quebec	Quebec	Sephardic Jewish	Turkish	Romani	Turkish	Mexico	Romani	China	Italy	7
*AP1S1* homozygous variants	IVS2-2A>G (splicing)	IVS2-2A>G (splicing)	356_365insG (stop codon)	c.364dupG (frameshif)	c.269T>C (missense)	c.346G>A (missense)	c.186T>G (stop codon)	c.269T>C (missense)	c.430-1G>A (frameshift)	c.256C>T (stop codon)	8
Exitus (*n*/tot)	3/5	3/3	0/1	0/1	3/3		0/1	2/2	0/2	0/1	11/19 (58%)
Multisystemic involvement (*n*/tot available data)											
Congenital enteropathy	5/5	3/3	1/1	1/1	3/3		1/1	2/2	1/1 *^(c)^*	1/1	18/18 (100%)
Dermatological involvement	3/3	3/3	1/1	1/1	0/3		0/0	2/2	2/2	1/1	13/16 (81%)
Deafness	4/4	2/3	1/1	1/1	0/3		0/0	2/2	2/2	1/1	13/17 (76%)
Impaired liver function	5/5	3/3	1/1	0/0	1/1		1/1	2/2	2/2	1/1	16/16 (100%)
Early growth deficit	3/3	0/0	1/1	1/1	1/1		1/1	0/0	1/1	1/1	9/9 (100%)
Neurological involvement (*n*/tot available data)											
Neurodevelopmental delay	4/4	1/1	1/1	0/0	0/0		1/1	1/1	2/2	1/1	11/11 (100%)
Hypotonia	3/3	1/1	1/1	1/1	0/0		0/0	0/2	1/2	1/1	8/11 (73%)
Neuropathy	5/5	1/1	0/1	0/1	0/0		0/0	1/1	2/2	0/1	9/12 (82%)
Cognitive impairment	3/3	2/2	1/1	1/1	0/0		0/0	1/1	2/2	1/1	11/11 (100%)
Seizures	0/3	0/2	0/1	0/1	1/3		1/1	0/2	2/2	1/1	5/16 (31%)
Brain MRI features (*n*/tot available data)											
Cortical atrophy	3/3	1/1	1/1	1/1	0/0		0/0	0/0	0/2	1/1 (progressive)	7/9 (78%)
Basal ganglia abnormalities	2/3	0/1	1/1	0/1	0/0		0/0	0/0	0/2	0/1 (+hippocampal involvement)	3/9 (33%)
Zinc treatment											
*n* treated patients	0	0	1	1	0		1	2	2	1	8

## Data Availability

The data that support the findings of this study are available on request from the corresponding author. The data are not publicly available due to privacy or ethical restrictions.

## Supplementary Materials

The following supporting information can be downloaded at: https://www.mdpi.com/article/10.3390/jcm15010106/s1, Supplementary Materials Video S1: Video-EEG seizure recording; Supplementary Materials Table S1: Extensive review of *AP1S1*-related MEDNIK syndrome reported features.
